# Anterior Cruciate Ligament Reconstruction With Hamstrings Autograft and Modified Lemaire Extra‐articular Tenodesis Using a Single Femoral Tunnel

**DOI:** 10.1002/atn2.70003

**Published:** 2026-06-22

**Authors:** Alessandro Carrozzo, Valerio Nasso, Silvia Cardarelli, Edoardo Monaco

**Affiliations:** ^1^ Dipartimento di Scienze della Vita, della Salute e delle Professioni Sanitarie Università degli Studi “Link Campus University” Rome Italy; ^2^ Sant’Andrea University Hospital Orthopedic and Trauma Surgery Unit La Sapienza University Rome Italy; ^3^ Tor Vergata University Hopsital Orthopedic and Trauma Surgery Unit Tor Vergata University Rome Italy

## Abstract

The addition of a lateral extra‐articular procedure to anterior cruciate ligament reconstruction has been shown to improve rotational stability and reduce graft failure rates, particularly in high‐risk patients. However, tunnel convergence remains a major concern in combined anterior cruciate ligament reconstruction and lateral extra‐articular procedure techniques, as it may compromise graft integrity and lead to failure. This technical note describes a technique for anterior cruciate ligament reconstruction using a tripled semitendinosus graft, maintaining its tibial attachment, combined with a modified Lemaire extra‐articular tenodesis. The femoral tunnel is created using an outside‐in drilling technique, allowing both the intra‐articular anterior cruciate ligament graft and the lateral extra‐articular tenodesis graft to be secured within the same tunnel using a single interference screw. This approach simplifies the procedure by eliminating additional fixation devices and reducing the risk of tunnel convergence, while maintaining bone stock and cost‐effectiveness.

## Graphical abstract

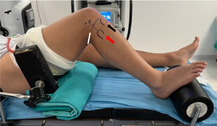

**VIDEO 1:** Demonstration of the outside‐in technique for anterior cruciate ligament and lateral extra‐articular tenodesis graft harvesting and preparation, femoral tunnel drilling, grafts passage and combined femoral fixation for a combined anterior cruciate ligament reconstruction and Modified Lemaire Extra‐Articular Tenodesis. Video content can be viewed at https://doi.org/10.1002/atn2.70003.

There is increasing evidence that the adding a lateral extra‐articular procedure (LEAP) to an anterior cruciate ligament reconstruction (ACLR) improves rotational stability and reduces graft failure rates, particularly in high‐risk patients such as those with high‐grade preoperative laxity, those with generalized hyperlaxity, those aiming to return to pivoting sports, and revision cases.^1^, ^2^, ^3^, ^4^ The modified Lemaire technique has been widely used to minimize failure rates and persistent rotational instability after ACLR.^3^, ^5^ However, tunnel convergence remains a significant concern in combined ACLR and LEAP procedures as it has been associated with graft damage and potential failure.^6^, ^7^, ^8^, ^9^ In addition, the need for multiple fixation devices and the relatively high incidence of symptomatic hardware associated with LEAP can further complicate these combined procedures.^10^

## SURGICAL TECHNIQUE

This article describes a surgical technique for ACLR with a tripled semitendinosus autograft (maintaining its distal attachment) combined with a modified Lemaire extra‐articular tenodesis. Through outside‐in drilling of the femoral tunnel, both graft constructs (intra‐articular and extra‐articular) are secured within the same femoral tunnel using 1 interference screw, thereby simplifying the procedure and minimizing hardware requirements (Video 1).

Pearls and pitfalls as well as advantages and disadvantages of this procedure are described in Tables 1 and 2.

**TABLE 1:** Pearls and Pitfalls

Pearls	Pitfalls
1. Preserve the tibial insertion of the semitendinosus to optimize vascularity and healing	1. Malpositioned tunnels may compromise both integrity and function of both ACL and LET grafts
2. Use an outside‐in technique for femoral drilling to avoid the risk of tunnel convergence and allow precise tunnel placement	2. Incorrect LET graft tensioning can lead to restricted range of motion or overconstraint of the knee
3. The lower part of the ITB should be released to ensure that the ITB can be closed at the end of the procedure	3. Fixation of the ACL and LET grafts in full extension can lead to screwdriver impingement with ITB
4. Pass the fascia lata strip deep to the LCL to utilize it as a pulley	4. Failure to secure the LET graft in near‐extension may result in fixation with the tibia externally rotated
5. Assess LET graft tension both in extension and in flexion before final fixation	

ACL, anterior cruciate ligament; ITB, iliotibial band; LCL, lateral collateral ligament; LET, lateral extra‐articular tenodesis.

**TABLE 2:** Advantages and Disadvantages

Advantages	Disadvantages
1. Single femoral fixation for both the intra‐articular and extra‐articular grafts reduces hardware requirements, enhancing cost‐effectiveness and surgical time	1. Technically demanding procedure, particularly for surgeons less experienced with outside‐in drilling
2. Maintaining the tibial insertion of the semitendinosus graft may promote better biologic healing	2. Risk of overconstraint if tensioning is not optimized, leading to restricted motion or tibial malrotation
3. Outside‐in femoral drilling avoids the risk tunnel convergence and potential graft damage	3. Requires an additional lateral incision to harvest the fascia lata strip for the modified Lemaire
4. Simplifies fixation by avoiding multiple tunnels and additional hardware (e.g., staples or separate screws)	4. Precise tunnel placement is critical; any error may compromise both the ACL and the LET graft
5. Combined graft construct provides enhanced rotational control and potentially reduces graft failure rates	5. Potential for symptomatic hardware remains if fixation devices are oversized or improperly placed

ACL, anterior cruciate ligament; LET, lateral extra‐articular tenodesis.

### Patients Positioning

**FIGURE 1:**
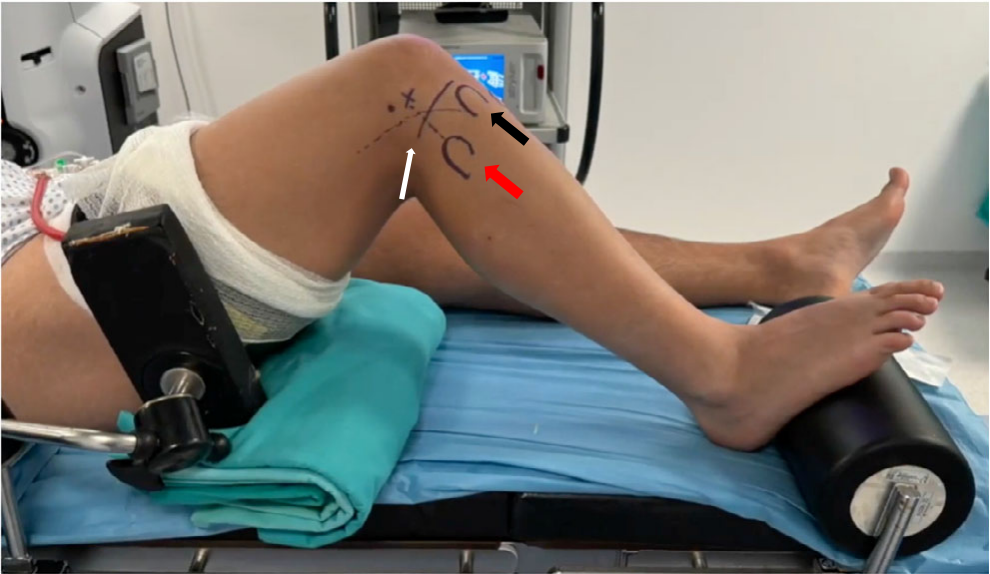
Patient in supine position. The right knee is flexed at 90° using a lateral support positioned at the height of a tourniquet and a foot support. The black arrow identifies Gerdy's tubercle; the red arrow identifies the head of the fibula; the white arrow identifies the knee joint line; the cross identifies the lateral femoral condyle; and the dot identifies the lateral femoralepicondyle.

The patient is positioned supine on the operating table with a well‐padded tourniquet applied proximally to the operative limb. A lateral post is placed at thigh level to provide countertraction, and the foot is supported in 90° flexion by a foot roll (Figure 1). Standard antiseptic preparation and draping is performed.

### Surgical Approach

Anterolateral and anteromedial portals are created. A thorough diagnostic arthroscopy is performed to assess for meniscal or chondral injuries, which are treated as necessary. The ACL remnant is selectively debrided on the femoral side to facilitate guide placement and ensure clear visualization of the femoral footprint, while preserving as much native tissue as possible.

### ACL Graft Harvesting and Preparation

**FIGURE 2:**
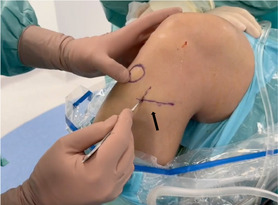
Patient in supine position with the right knee flexed at 90°. Incision at the pes anserinus. The circle identifies the tibial tuberosity and the black arrow identifies the course of the pes anserinus tendons.

**FIGURE 3:**
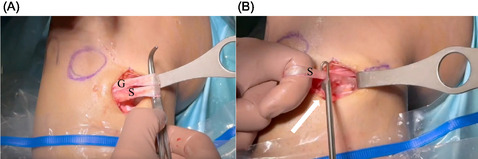
Patient in supine position with the right knee flexed at 90°. The semitendinosus (S) and gracilis (G) tendons are identified (A) and the semitendinosus tendon is harvested using an open‐ended tendon stripper (B) while preserving its distal tibial insertion.

**FIGURE 4:**
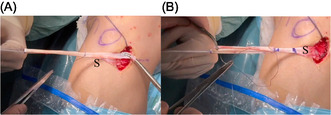
Patient in supine position with the right knee flexed at 90°. The steps to triple the semitendinosus tendon. (A) The tendon (S) is duplicated. (B) The tendon (S) is tripled. Vycril 0 thread is used to perform the sutures.

A 3‐ to 4‐cm vertical incision is made over the pes anserinus to allow identification of the semitendinosus tendon (Figure 2). The tendon is carefully harvested using an open‐ended tendon stripper, preserving its distal tibial attachment (Figure 3). The harvested tendon is then prepared on by tripling to achieve the required diameter and length for ACL reconstruction. The femoral free end of the graft is whipstitched to allow graft passage and tensioning (Figure 4).

### LET Graft Harvesting and Preparation

**FIGURE 5:**
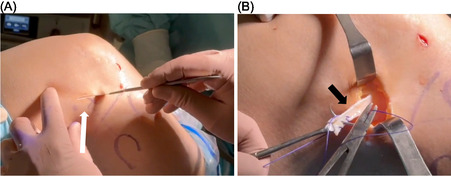
Patient in supine position with the right knee flexed at 90°. Longitudinal incision (white arrow) of 5 to 6 cm is made proximal to the lateral epicondyle (A), the cross identifies the lateral femoral condyle, and the dot identifies the lateral femoral epicondyle; a strip of fascia lata (black arrow) is harvested with No. 0 FiberWire, maintaining its distal attachment to Gerdy's tubercle, 1 cm wide and 8 to 10 cm long (B).

A 5‐ to 6‐cm longitudinal incision is made just proximal to the lateral epicondyle, exposing the iliotibial band (ITB) (Figure 5A). A 1‐cm‐wide strip of fascia lata is harvested, extending approximately 8 to 10 cm proximally while maintaining its distal attachment to Gerdy's tubercle. A whip stitch with a No. 0 FiberWire (Arthrex Inc., Naples, FL, USA) is placed at the free end to facilitate handling, shuttling, and the subsequent tensioning (Figure 5B).

### Tunnels Preparation

**FIGURE 6:**
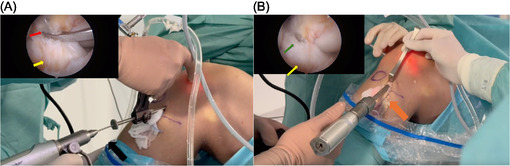
Patient in supine position with the right knee flexed at 90°. Preparation of the tibial tunnel using a standard tibial guide for the anterior cruciate ligament (ACL), first with a guide pin (black arrow) (A) and then with a reamer (orange arrow) of increasing diameter until the desired diameter is achieved (B). Arthroscopic view (A,B) through the anterolateral portal of the ACL remnant (yellow arrow), guide wire (red arrow), and reamer (green arrow). (ACL, anterior cruciate ligament.)

The tibial tunnel is then prepared using a standard ACL tibial guide. A guide pin is placed in the center of the native ACL footprint (Figure 6A) and reamed to the appropriate size (Figure 6B).

**FIGURE 7:**
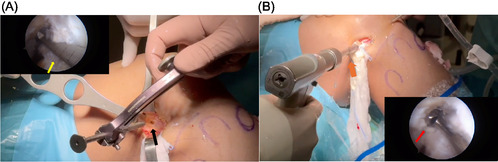
Patient in supine position with the right knee flexed at 90°. Preparation of the femoral tunnel using an outside‐in drilling technique, first with a guide pin positioned proximally and posterior to the lateral epicondyle (black arrow) (A) and then with a reamer (orange arrow) of increasing diameter until the desired diameter is reached (B). Arthroscopic view through the anterolateral portal of the guide positioned on the lateral femoral condyle (yellow arrow) and reamer (red arrow).

For femoral tunnel creation, an outside‐in drilling technique is utilized. A guide pin is inserted through the lateral incision, positioned proximal and posterior to the lateral epicondyle, and directed toward the anatomic ACL footprint under arthroscopic guidance (Figure 7B). The tunnel is then reamed sequentially to match the graft diameter (typically 8‐9 mm) (Figure 7B).

A No. 2 passing suture is inserted through the femoral tunnel into the joint, retrieved from the tibial tunnel, and designated as the ACL graft shuttle. Another No. 2 passing suture is inserted through the femoral tunnel and retrieved through the anteromedial arthroscopic portal to serve as the lateral extra‐articular tenodesis (LET) graft shuttle.

### Graft Passages and Fixation

**FIGURE 8:**
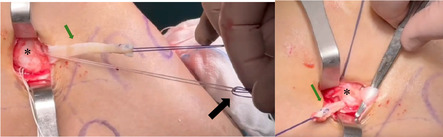
Patient in supine position with the right knee flexed at 90°. The fascia lata (green arrow) is passed under the LCL (asterisk) through a loop (black arrow) that is first passed under the LCL. (LCL, lateral collateral ligament.)

**FIGURE 9:**
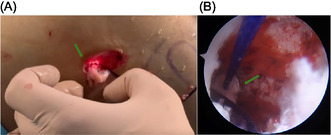
Patient in supine position with the right knee flexed at 90°. The passage of the fascia lata (green arrow) through the femoral tunnel (A); arthroscopic view through the anteromedial portal of the end of the fascia lata (green arrow) in the the femoral tunnel (B).

**FIGURE 10:**
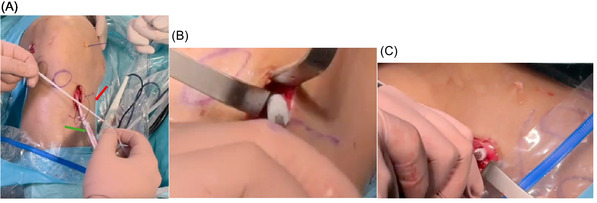
Patient in supine position with the right knee flexed at 90°. The graft (green arrow) is passed through the tibial tunnel with a suture loop (red arrow) previously inserted into the femoral and tibial tunnels (A); the graft is then fixed on the tibial side with an interference screw (b) with the knee flexed at 90° and on the femoral sides with an interference screw (C) with the knee in near extension.

Then, the fascia lata strip is passed deep to the lateral collateral ligament (LCL) (Figure 8) and into the femoral tunnel (Figure 9). Then, maintaining tension to the LET graft wires from the anteromedial portal, the tripled semitendinosus graft is passed through the tibial tunnel into the joint and retrieved through the femoral tunnel (Figure 10A).

With the knee positioned at 90°, a Nitinol guide wire is inserted into the tibial tunnel; an interference screw (Biocomposite; Arthrex) is then inserted into the tibial tunnel with tension applied to the ACL graft (Figure 10B). A Nitinol guide wire is inserted into the femoral tunnel, followed by placement of an interference screw with the knee in near extension (Figure 10C). Simultaneous tension is applied to both the ACL and LET grafts, effectively securing the intra‐articular ACL graft and the extra‐articular fascia lata strip within the same tunnel.

### Postoperative Rehabilitation

Postoperatively, patients are encouraged to bear weight as tolerated with crutches for comfort. Early rehabilitation focuses on regaining full extension, quadriceps activation, and progressive range of motion. Return to sports is guided by functional milestones, typically allowing nonpivoting sports at 4 months, pivoting noncontact sports at 6 months, and full return to pivot and contact sports at 8 to 9 months.

## DISCUSSION

The described technique addresses some of the challenges frequently encountered in combined ACLR and LEAP procedures: tunnel convergence and the need for multiple fixation devices potentially leading to symptomatic hardware.

By using outside‐in drilling for the ACL femoral tunnel, the surgeon gains a more versatile angle of approach, allowing accurate placement of a single femoral tunnel that will accommodate both the intra‐articular and extra‐articular grafts.

To minimize the risk of ACL and LET tunnel convergence, different techniques have been proposed. A study analyzing postoperative computed tomography scans in patients undergoing combined ACL reconstruction with anteromedial drilling and modified Lemaire with femoral fixation through independent bony tunnels highlighted the importance of tunnel orientation in preventing convergence.^11^ In 52 cases, tunnel collision was observed in 15.4% of cases, and in 26.9% of cases, the shortest distance between the tunnels was less than 5 mm. Importantly, when the LET tunnel was drilled with an anterior tilt of less than 15°, convergence was inevitable. This supports the rationale for outside‐in femoral drilling in ACL reconstruction, as it provides better control over tunnel orientation, thereby reducing the risk of convergence. Hopper et al.^6^ proposed utilizing outside‐in femoral drilling for ACL reconstruction, and the fixation of the modified Lemaire with a knotless suture anchor fixed through a small 2.6‐mm tunnel, which allows for precise directional control and substantially reduces the risk of ACL graft collision. Onlay fixation for the LET graft has shown outcomes comparable to intratunnel fixation.^12^ However, metallic staples, the most commonly used hardware for onlay fixation, are associated with a relatively high incidence of lateral pain, symptomatic hardware, and subsequent hardware removal.^10^

Inserting the LET graft into the same tunnel alongside the ACL graft permits a single interference screw to secure both constructs. Various combined ACL and LEAP techniques have already adopted this approach it to eliminate the need for additional hardware and accessory bone tunnels.^13^, ^14^

Particularly, combined ACLR and anterolateral ligament reconstruction (ALLR) techniques have shown excellent rotational control, reduced graft rupture rates, and minimized hardware‐related complications.^15^, ^16^

Although the surgeon must carefully assess tensioning to avoid overconstraint, especially in terminal extension, the single‐femoral‐tunnel method is reproducible and cost‐effective. A recent analysis by Giusto et al.^17^ compared LET and ALLR for primary ACL reconstruction, evaluating clinical outcomes, graft failure rates, and associated costs. Their systematic review of 22 studies involving 2505 knees found no significant difference in ACL graft failure rates between LET and ALLR (2.9% vs 3.2%, *P* = .690). However, LET resulted in slightly higher quality‐adjusted life‐years and shorter operative time. Cost analysis showed that LET ($1015) was more affordable than both autograft ($1295) and allograft ($3068) ALLR.

The use of a technique with a single femoral tunnel, whether for ACL reconstruction combined with a LET or with an ALLR, provides a more cost‐effective solution for performing LEAPs alongside ACLR.

In conclusion, the use of outside‐in femoral drilling for ACL reconstruction with a tripled semitendinosus graft, combined with modified Lemaire extra‐articular tenodesis secured within the same femoral tunnel, provides a simple and reliable technique for treating both anteroposterior and anterolateral rotational instability in primary and revision ACL surgery. This approach preserves bone stock, minimizes the risk of tunnel convergence, and remains cost‐effective.

## DISCLOSURES

The author (E.M.) declares the following financial interests/personal relationships which may be considered as potential competing interests: E.M. reports a relationship with Arthrex Inc. that includes consulting or advisory. The other authors (A.C., V.N., S.C.) declare that they have no known competing financial interests or personal relationships that could have appeared to influence the work reported in this paper.

## FUNDING

Open access publishing facilitated by Universita degli Studi di Roma La Sapienza, as part of the Wiley ‐ CRUI‐CARE agreement.

## References

[1] Bosco F , Giustra F , Masoni V et al. Combining an anterolateral complex procedure with anterior cruciate ligament reconstruction reduces the graft reinjury rate and improves clinical outcomes: A systematic review and meta‐analysis of randomized controlled trials. Am J Sports Med. 2024;52:2129‐2147. 38353002 10.1177/03635465231198494

[2] Sonnery‐Cottet B , Carrozzo A . Lateral extra‐articular tenodesis and anterolateral procedures. Clin Sports Med. 2024;43:413‐431. 38811119 10.1016/j.csm.2023.08.008

[3] Getgood AMJ , Bryant DM , Litchfield R , et al. Lateral extra‐articular tenodesis reduces failure of hamstring tendon autograft anterior cruciate ligament reconstruction: 2‐Year outcomes from the STABILITY study randomized clinical trial. Am J Sports Med. 2020;48:285‐297. 31940222 10.1177/0363546519896333

[4] Sonnery‐Cottet B , Saithna A , Cavalier M , et al. Anterolateral ligament reconstruction is associated with significantly reduced ACL graft rupture rates at a minimum follow‐up of 2 years: A prospective comparative study of 502 patients from the SANTI Study Group. Am J Sports Med. 2017;45:1547‐1557. 28151693 10.1177/0363546516686057

[5] Sherman SL , Calcei J , Ray T , et al. ACL Study Group presents the global trends in ACL reconstruction: Biennial survey of the ACL Study Group. J ISAKOS. 2021;6:322‐328. 34272329 10.1136/jisakos-2020-000567

[6] Hopper GP , El Helou A , Philippe C , Campos JP , Vieira TD , Sonnery‐Cottet B . How to avoid knee tunnel convergence when performing a modified lemaire extra‐articular tenodesis. Arthrosc Tech. 2022;11:e1111‐e1115. 35782851 10.1016/j.eats.2022.02.019 PMC9244758

[7] Mesnier T , Cavaignac M , Marot V , Reina N , Cavaignac E . Knee anterolateral ligament reconstruction with knotless soft anchor: Shallow fixation prevents tunnel convergence. Arthrosc Tech. 2022;11:e511‐e516. 35493031 10.1016/j.eats.2021.11.024 PMC9051614

[8] Smeets K , Bellemans J , Lamers G , et al. High risk of tunnel convergence during combined anterior cruciate ligament and anterolateral ligament reconstruction. Knee Surg Sports Traumatol Arthrosc. 2019;27:611‐617. 30298415 10.1007/s00167-018-5200-3

[9] Jaecker V , Ibe P , Endler CH , Pfeiffer TR , Herbort M , Shafizadeh S . High risk of tunnel convergence in combined anterior cruciate ligament reconstruction and lateral extra‐articular tenodesis. Am J Sports Med. 2019;47:2110‐2115. 31194569 10.1177/0363546519854220

[10] Heard M , Marmura H , Bryant D , et al. No increase in adverse events with lateral extra‐articular tenodesis augmentation of anterior cruciate ligament reconstruction—Results from the stability randomized trial. J ISAKOS. 2023;8:246‐254. 36646169 10.1016/j.jisako.2022.12.001

[11] Perelli S , Erquicia JI , Ibañez M , et al. Evaluating for tunnel convergence in anterior cruciate ligament reconstruction with modified lemaire tenodesis: What is the best tunnel angle to decrease risk? Arthroscopy. 2020;36:776‐784. 31864816 10.1016/j.arthro.2019.08.042

[12] Behrendt P , Fahlbusch H , Akoto R , et al. Comparison of onlay anchor fixation versus transosseous fixation for lateral extra‐articular tenodesis during revision ACL reconstruction. Orthop J Sports Med. 2023;11:23259671231166380. 37213658 10.1177/23259671231166380 PMC10196542

[13] Moro R , Thá VC , Ruedas VR , Tauchmann R , Dantas GM , Filho MD . Description of the anterior cruciate ligament reconstruction technique with mini‐lemaire type anterolateral tenodesis through a single femoral tunnel. Rev Bras Ortop (Sao Paulo). 2024;59:e313‐e317. 38606133 10.1055/s-0044-1779326 PMC11006511

[14] Sonnery‐Cottet B , Daggett M , Helito CP , Fayard JM , Thaunat M . Combined anterior cruciate ligament and anterolateral ligament reconstruction. Arthrosc Tech. 2016;5:e1253‐e1259. 28149722 10.1016/j.eats.2016.08.003 PMC5263705

[15] Sonnery‐Cottet B , Pioger C , Vieira TD et al. Combined ACL and anterolateral reconstruction is not associated with a higher risk of adverse outcomes: Preliminary results from the SANTI randomized controlled trial. Orthop J Sports Med. 2020;8:2325967120918490. 32490026 10.1177/2325967120918490 PMC7238835

[16] Thaunat M , Clowez G , Saithna A , et al. Reoperation rates after combined anterior cruciate ligament and anterolateral ligament reconstruction: A series of 548 patients from the SANTI Study Group with a minimum follow‐up of 2 years. Am J Sports Med. 2017;45:2569‐2577. 28610433 10.1177/0363546517708982

[17] Giusto JD , Cohen D , Dadoo S , et al. Lateral extra‐articular tenodesis may be more cost‐effective than independent anterolateral ligament reconstruction: A systematic review and economic analysis. J ISAKOS. 2024;9:689‐698. 38604570 10.1016/j.jisako.2024.04.004

